# Gas Permeability, Fractional Free Volume and Molecular Kinetic Diameters: The Effect of Thermal Rearrangement on *ortho*-hydroxy Polyamide Membranes Loaded with a Porous Polymer Network

**DOI:** 10.3390/membranes12020200

**Published:** 2022-02-09

**Authors:** Cenit Soto, Edwin S. Torres-Cuevas, Laura Palacio, Pedro Prádanos, Benny D. Freeman, Ángel E. Lozano, Antonio Hernández, Bibiana Comesaña-Gándara

**Affiliations:** 1 Surfaces and Porous Materials (SMAP), Associated Research Unit to CSIC, Facultad de Ciencias, University of Valladolid, Paseo Belén 7, E-47011 Valladolid, Spain; marveliacenit.soto@uva.es (C.S.); laura.palacio@uva.es (L.P.); ppradanos@uva.es (P.P.); lozano@ictp.csic.es (Á.E.L.); 2 Institute of Sustainable Processes (ISP), E-47011 Valladolid, Spain; 3 McKetta Department of Chemical Engineering, Texas Materials Institute, The University of Texas at Austin, 200 E Dean Keeton St., Austin, TX 78712, USA; edwinstorres@utexas.edu (E.S.T.-C.); freeman@che.utexas.edu (B.D.F.); 4 Institute for Polymer Science and Technology (ICTP-CSIC), Department of Macromolecular Chemistry, Juan de la Cierva 3, E-28006 Madrid, Spain; 5 IU CINQUIMA, University of Valladolid, Paseo Belén 5, E-47011 Valladolid, Spain

**Keywords:** mixed-matrix membranes, gas separation, hydrogen separation, thermal rearrangement, porous polymer network

## Abstract

Mixed-matrix membranes (MMMs) consisting of an *ortho*-hydroxy polyamide (HPA) matrix, and variable loads of a porous polymer network (PPN) were thermally treated to induce the transformation of HPA to polybenzoxazole (β-TR-PBO). Two different HPAs were synthesized to be used as a matrix, 6FCl-APAF and tBTpCl-APAF, while the PPN used as a filler was prepared by reacting triptycene and trifluoroacetophenone. The permeability of He, H_2_, N_2_, O_2_, CH_4_ and CO_2_ gases through these MMMs are analyzed as a function of the fraction of free volume (FFV) of the membrane and the kinetic diameter of the gas, allowing for the evaluation of the free volume. Thermal rearrangement entails an increase in the FFV. Both before and after thermal rearrangement, the free volume increases with the PPN content very similarly for both polymeric matrices. It is shown that there is a portion of free volume that is inaccessible to permeation (occluded volume), probably due to it being trapped within the filler. In fact, permeability and selectivity change below what could be expected according to densities, when the fraction of occluded volume increases. A higher filler load increases the percentage of inaccessible or trapped free volume, probably due to the increasing agglomeration of the filler. On the other hand, the phenomenon is slightly affected by thermal rearrangement. The fraction of trapped free volume seems to be lower for membranes in which the tBTpCl-APAF is used as a matrix than for those with a 6FCl-APAF matrix, possibly because tBTpCl-APAF could approach the PPN better. The application of an effective medium theory for permeability allowed us to extrapolate for a 100% filler, giving the same value for both thermally rearranged and non-rearranged MMMs. The pure filler could also be extrapolated by assuming the same tendency as in the Robeson’s plots for MMMs with low filler content.

## 1. Introduction

Mixed-matrix membranes (MMMs) have emerged as promising materials for gas separation in membrane technology. MMMs consist of a mixture of organic or inorganic porous materials as a dispersed phase (filler) within a polymeric matrix as a continuous phase [1,2,3,4]. MMMs benefit from the potential synergy between the polymeric matrix and the fillers, which enhances the properties of MMMs compared to those of the pure polymer [5] and exhibits a superior performance in terms of gas permeability and/or selectivity [6].

The inorganic and organic materials used as fillers should have a unique structure, surface chemistry and mechanical strength. Overall, MMMs should increase permeability while at least maintaining selectivity by introducing fillers into the polymeric matrix, as a result of a more or less selective increase in diffusion and/or solubility coefficients [7].

There are multiple factors that, during the manufacture of MMMs, can induce non-ideal effects, for example, interfacial defects caused by particle sedimentation or agglomeration, or the migration of filler particles to the surface, especially when the load of fillers is high [8]. Moreover, the compatibility between dispersed and continuous phases is an important factor to consider [9]. Indeed, an effective contact between the two phases is necessary to avoid any gaps between them that could block access to the pores. The ideal morphology of MMMs involves no defects in the polymer–particle interface and must guarantee gas transport through the dispersed phase rather than through the continuous phase. Indeed, the dispersed phase, accepting that it is uniformly distributed, is always encapsulated by an “interface” (region between inorganic fillers and polymer matrix) with properties different from both the dispersed and continuous phases [10].

**Figure 1:**
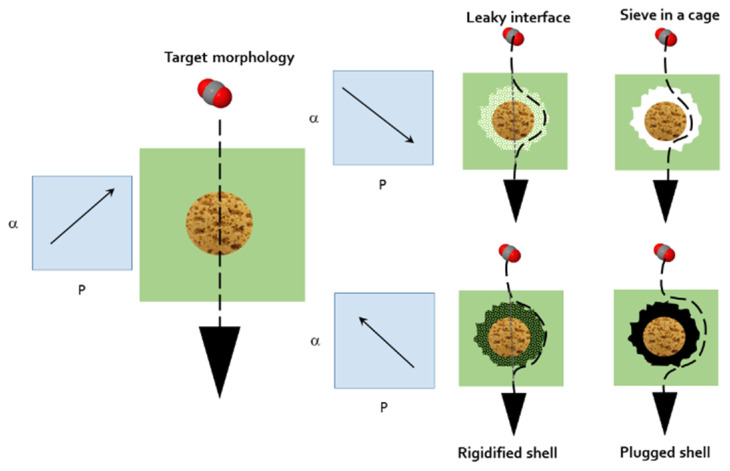
Schematic representation of possible defects formed at the polymer–particle interface. The corresponding expectable selectivity versus permeability behaviors are shown by the arrows.

In fact, the manufacture of an ideal MMM is a complex process due to the formation of defects at the polymer–particle interface that may arise due to a weak polymer–particle adhesion, caused by the difference in properties between both phases. These defects at the interface between the dispersed and continuous phases can affect membrane properties, mainly the separation performance of the membrane as shown schematically in Figure 1. 

A low adhesion between the dispersed and the continuous phases could lead to the formation of non-selective voids in the interfacial region. Other causes contributing to the formation of interfacial voids are the alteration of the polymer packing in the vicinity of the dispersed particles, the repulsive force between the two phases and the different coefficients of thermal expansion [8]. Moreover, interfacial voids or sieves-in-a-cage can be attributed to the de-wetting of the polymeric chains on the external surface of the particles [7]. Moore and Koros [10] observed that solvent evaporation, thermal effects and the resulting stresses at the polymer–filler interface cause defects such as interface void formation. The formation of these defects allows the gases to pass and, hence, deteriorates the apparent selectivity and increases the permeability of MMMs. These factors can give an incomplete detachment of the polymeric matrix and the filler, giving rise to leaky interfaces.

A rigidified polymer layer around the inorganic fillers occurs when the polymer matrix chains are in direct contact with the filler surface. They are rigidified as compared with the bulk polymer chains, which reduces the free volume and is related to a uniform tension around the particles [11]. Moore and Koros [10] hypothesized that polymer rigidification enhances the diffusive selectivity and decreases membrane permeability. Rigidified interfaces can be caused by particle pore blockage or clogging that can be generated by the presence of sorbent, solvent traces, a contaminant or a minor component in the feed gas, before, during and after the manufacturing of MMMs [4]. If the pores are completely blocked, the gas cannot pass through the particle fillers, and no enhancement in selectivity over the neat polymer is reached, as in the case of MMMs filled with non-porous particles.

On the other hand, MMMs can eventually undergo thermal transposition processes such as thermal rearrangement (TR) at a high temperature [12,13,14], which can further increase gas permeabilities. For instance, thermal treatment of a precursor poly(*o*-hydroxyamide) produces polybenzoxazole (PBO) structures with outstanding transport properties for the separation of gases (TR polymers) [15]. As already mentioned, the manufacture of high-performance MMMs depends on the appropriate filler selection to prevent the formation of non-selective voids caused by the low polymer–filler affinity [3,16]. In this sense, metal–organic frameworks (MOFs) with high surface area and porosity [17], covalent organic frameworks (COFs) or porous aromatic frameworks (PAFs) with large surface areas and thermal stability [18,19], and hypercrosslinked polymers (HCPs) with significant potential for CO_2_ adsorption [20], have been successfully used as fillers in MMMs for gas separation. Recently, some novel materials based on porous polymer networks (PPNs) [21] have shown to be good candidates to be used as fillers to prepare MMMs with promising gas permeabilities [22].

Despite the potential of MMMs, there is a limited knowledge of the TR-MMMs gas separation performance. Our aim here is to correlate permeability (and consequently, selectivity) to free volume and its changes after thermal rearrangement. This work analyzes these correlations and proposes a simple model for permeability in terms of free volume fraction with two hydroxy polyamides (HPAs) and their thermally rearranged β-TR counterparts.

## 2. Materials and Methods

The polymers and MMM membranes synthetized at the SMAP group and tested elsewhere [14,23,24] were used. Key data on the synthesis and manufacture of these polymers and MMMs are given below to contribute to readability.

### 2.1. Polymer Synthesis

**Figure 2:**
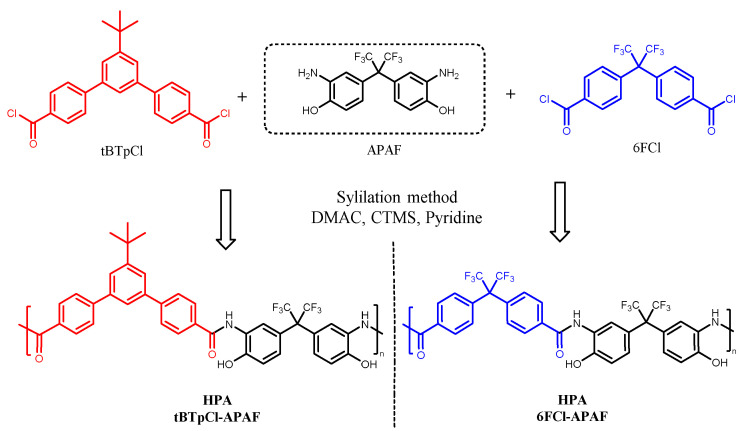
Synthesis scheme of the two precursor HPAs used as polymer matrix in the manufacture of the MMMs of this work.

Two HPAs, 6FCl-APAF and tBTpCl-APAF, synthetized by the low-temperature polycondensation process with activation of the diamines by in situ silylation [25], whose schemes are shown in Figure 2, were used as a polymeric matrix or continuous phase. In brief, 2,2-Bis(3-amino-4-hydroxyphenyl)hexafluoropropane (APAF, CymitQuimica, Barcelona, Spain, CAS #83558-87-6) was mixed with 2,2′-bis(4-carboxy-phenyl)hexafluoropropane diacid chloride (6FCl, synthetized according to Smith et al. 2014 [26]) and 5′-tertbutyl-*m*-terphenyl-4,4″-dichloride acid (tBTpCl) synthetized as described in a previous work [23] with stoichiometric ratios in N,N-Dimethylacetamide (DMAC, CymitQuimica, CAS #127-19-5), before the addition at 0 °C of chlorotrimethylsilane (CTMS, CymitQuimica, CAS #75-77-4), anhydrous pyridine (CymitQuimica, CAS #110-86-1) and 4-(Dimethylamino)pyridine (DMAp, CymitQuimica, CAS #1122-58-3), under nitrogen stream overnight. 

### 2.2. Filler Synthesis

**Figure 3:**
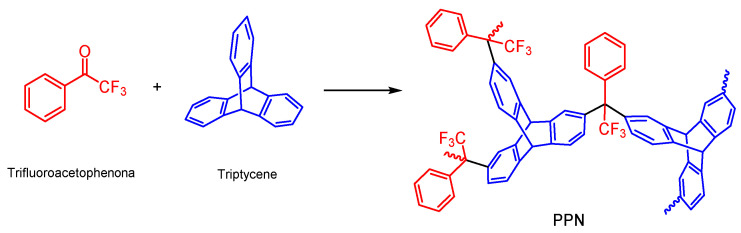
Reaction scheme of the PPN used as filler in the manufacture of the MMMs of this work.

A porous polymer network (PPN), used as a dispersed phase, was synthetized prior to this work by reacting triptycene and 2,2,2-trifluoroacetophenone, according to the methodology described by Lopez-Iglesias et al. [27] (Figure 3).

### 2.3. Manufacture of MMMs 

MMMs were prepared using the solution casting method. A suitable amount of the selected HPA was dissolved in tetrahydrofuran. Simultaneously, the corresponding amount of the PPN (20 or 30%) was well dispersed in tetrahydrofuran by sonication for 20 min and was then added to the previous polymer solution. The mixture obtained was cast onto a glass plate and slowly heated for solvent evaporation up to 180 °C under vacuum conditions. The precursor HPA-based MMMs were subjected to a thermal rearrangement at 375 °C for 15 min under N_2_ atmosphere to obtain the corresponding porous and rigid TR-PBO-MMM.

Both polymer matrices, the PPN used as filler and all MMMs therefore obtained were properly characterized in previous works [14,23,24].

## 3. Fractional Free Volume

The fractional free volume, FFV, is defined as:

(1) $$FFV^{i}=\frac{Vi-V_{0}^{i}}{V^{i}}\;i=HPA,\;PPN$$

where $V^{i}$ is the total specific volume and $V_{0}^{i}$ is the specific skeletal volume of the phase. The skeletal volume for HPA and the PPN can be evaluated from their Van der Waals volumes as follows:

(2) $$V_{0}^{i}\approx 1.3\;V_{w}^{i}\;i=HPA,\;PPN$$

where $V_{w}^{HPA}$ and $V_{w}^{PPN}$ could be evaluated by the Bondi group theory with all its drawbacks [28]. However, the parameters were herein calculated via molecular modeling using the Materials Studio software (BIOVIA, San Diego, CA, USA). This allowed us to evaluate $V_{0}^{HPA}$ and $V_{0}^{PPN}$ using Equation (2).

The HPA’s specific volume $V^{HPA}$ can be obtained from densities $\left(V^{HPA}=1/\rho^{HPA}\right)$ as measured by following Archimedes’ principle in a CP225 Analytical Balance from Sartorius (Sartorius, Göttingen, Germany) equipped with a density measurement kit. The samples were weighed in air and in high pure iso-octane at room temperature. The average density from seven samples was obtained according to Equation (3):

(3) $${}^{\rho^{HPA}=\rho_{C_{8}H_{18}}\frac{W_{air}}{W_{air}-W_{C_{8}H_{18}}}}$$

where $\rho_{C_{8}H_{18}}$ corresponds to the iso-octane’s density, $W_{air}$ corresponds to the sample weight and $W_{C_{8}H_{18}}$ stands for the weight of the sample when submerged in iso-octane. Finally, Equations (1) and (2) allow for the evaluation of the FFV for HPA.

The PPN’s specific volume can be evaluated as the sum of its skeletal specific volume $V_{0}^{PPN}$ plus the specific volume within the PPN pores $V_{p}^{PPN}$:

(4) $$V^{PPN}=V_{0}^{PPN}+V_{p}^{PPN}$$

$V_{0}^{PPN}$ is measured by gas pycnometry with an AccuPyc 1330 V2.04N (Micromeritics Instrument Corporation, Norcross, GA, USA). Other more indirect methods can be used [14] but are evidently subject to a high potential for errors. Here, the skeletal volume is determined by gas displacement using the volume–pressure relationship of Boyle’s law. An inert gas, helium, is used as the displacement medium. The sample is placed in a sealed cup of a known volume (2.5 cm^3^). Gas is introduced to the sample chamber and then expanded into a second empty chamber with a known volume. The pressure observed after filling the sample cell and the pressure discharged into the expansion chamber are measured, and then the volume is calculated. Density is determined by dividing the sample weight by the volume measured. Manufacturers claim that the precision of the AccuPyc apparatus is typically within ±0.01% of the nominal full-scale cell chamber volume. Reproducibility is guaranteed to be ±0.02% of the nominal full-scale volume on clean, dry, thermally equilibrated samples using helium in the 15 to 35 °C range with an accuracy of 0.03%.

Furthermore, $V_{P}^{PPN}$ is measured by CO_2_ adsorption-desorption at 0 °C (273 K) in the volumetric device Nova 4200 (Quantachrome, Boynton Beach, FL, USA). The samples were degassed at 125 °C for 18 h under vacuum before the initiation of the CO_2_ adsorption measurements. Equations (1), (2) and (4) allow for the evaluation of the PPN’s FFV.

(5) $$FFV^{MMM}=\phi \;FFV^{PPN}+\left(1-\phi \right)\;FFV^{HPA}$$

This equation correlates the fraction of free volume in terms of *ϕ*, the fraction of filler (PPN) and assumes that there is not any significant interaction between filler and matrix. 

## 4. Permeability Versus Kinetic Diameters

The dependence of diffusivities (and permeability) on the kinetic diameter and free volume, which has been studied extensively in the literature, was herein analyzed to evaluate the contributions to free volume caused by filler–matrix interactions. A deep revision on this topic was made by Matteucci et al. [29] and more recently by Thornton et al. [30], who proposed a diffusivity *D* versus fractional free volume *f* (or FFV) given by the relationship:

(6) $$D=\alpha e{\;}^{\beta f}$$

This equation is based on the Doolittle [31] equation, and it was shown to fit better the experimental results [30]. Thornton et al. showed that for membranes where diffusion controls transport, the permeability follows a similar dependence on free volume:

(7) $$P=SD=Ae^{Bf}$$

In fact, it can be assumed that this equation holds when solubility is almost independent of the FFV or depends, like diffusivity, exponentially on *f*.

Several models [32,33] based on a supposed linear dependence of the diffusion activation energy with the transversal area of the penetrant, admit a relationship of the logarithm of *D* with the square of the kinetic diameter, *δ*, [34,35]. Less frequently, relationships with linear or cubic powers of *δ* [29] have also been considered. Sometimes piecewise fittings have been used for the sake of comparison [36]. Here, a quadratic dependence of *B* (Equation (7)) would be needed to fit the results (Equation (8)).

(8) $$B=a+b\delta +c\delta^{2}$$

Combining Equation (8) with Equation (7), we obtain:

(9) $$\ln P=\left[\ln A+af\right]+\left[bf\right]\delta +\left[cf\right]\delta^{2}$$

**Figure 4:**
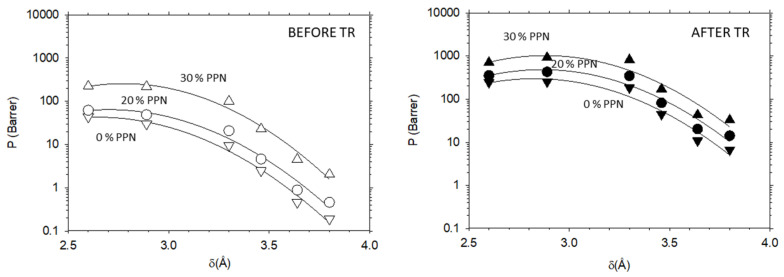
Permeability as a function of the gas kinetic diameters before thermal rearrangement (**left**) and after thermal rearrangement (**right**) for the membranes with 6FCl-APAF matrix. Inverted triangles (▽), circles (○) and triangles (△) represent 0%, 20% and 30% PPN loading, respectively.

**Figure 5:**
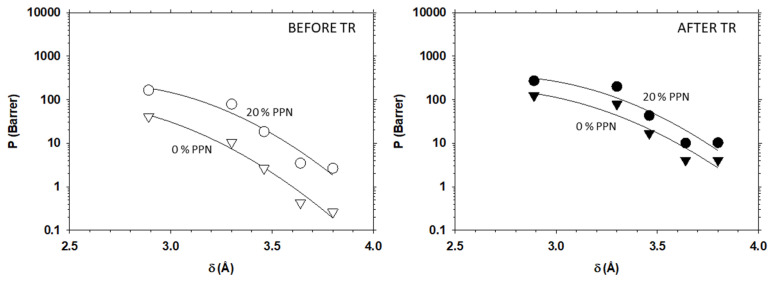
Permeability as a function of the gas kinetic diameters before thermal rearrangement (**left**) and after thermal rearrangement (**right**) for the membranes with tBTpCl-APAF matrix. Inverted triangles (▽) and circles (○) represent 0% and 20% PPN loading, respectively.

Therefore, permeability depends on the gas kinetic diameter as shown in Figure 4 and Figure 5 for He, H_2_, N_2_, O_2_, CH_4_ and CO_2_ gases. The lines, shown in these figures, fit the quadratic equation (Equation (9)) satisfactorily with fitted parameters as shown in Table 1 and Table 2. Here, the kinetic diameters given by Breck [29,37] were used.

Some data of gas permeabilities shown in Figure 4 and Figure 5 have been previously reported by Soto et al. [14,23,24]. 

In these figures, it is clearly shown that permeability increases for all gases with PPN content, before and after thermal rearrangement, and decreases for gases with higher kinetic diameters. Moreover, it can be also observed that permeabilities are only slightly higher for tBTpCl-APAF than for 6FCl-APAF.

## 5. Permeability and Selectivity versus PPN Content

**Figure 6:**
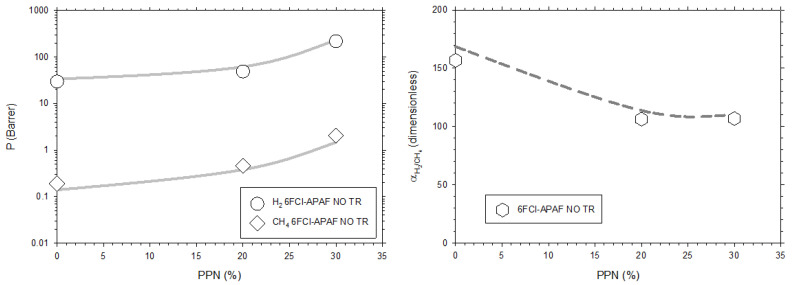
Permeability of H_2_ and CH_4_ as a function of the PPN content (**left**) and the corresponding selectivity (**right**). Results for the MMMs containing 6FCl-APAF as matrix without thermal rearrangement are shown. Lines correspond to the fittings shown on Table 1 and Table 2.

The permeability for a given gas as a function of the PPN load increases, while all selectivities decrease, when the PPN content increases. As an example, H_2_ and CH_4_ permeabilities and the corresponding selectivity for membranes in which the 6FCl-APAF polymeric matrix is used, are shown in Figure 6.

Hydrogen is especially relevant in many industrial applications and is a valuable green energy source [38]. Today, the production of H_2_ basically relies on the decomposition of CH_4_, in which the separation of H_2_ from H_2_/CH_4_ mixtures is of primary importance [38,39]. Moreover, an efficient separation process should make it possible for the two substances to be routed through the established and extensive natural gas grid together and then isolated from one another at their final destination [40].

According to Equation (9), the selectivity $\alpha_{1,2}=P_{1}/P_{2}$ for the 1–2 gas pair can be calculated according to Equation (10):

(10) $$\ln\alpha_{1,2}=bf\left(\delta_{1}-\delta_{2}\right)+cf\left(\delta_{1}^{2}-\delta_{2}^{2}\right)=f\left(\delta_{1}-\delta_{2}\right)\;\left[b+c\left(\delta_{1}-\delta_{2}\right)\right]$$

assuming that *b* and *c* do not depend on the gas. Consequently, selectivity $\alpha_{1,2}>1$, i.e., gas 1 would be enriched in the permeate, if:

(11) $$f\left(\delta_{1}-\delta_{2}\right)\;\left[b+c\left(\delta_{1}+\delta_{2}\right)\right]>0$$

If $\delta_{1}<\delta_{2}$, it must be:

(12) $$b+c\;\left(\delta_{1}+\delta_{2}\right)<0\Leftrightarrow c<\frac{b}{\delta_{1}+\delta_{2}}$$

Otherwise, if $\delta_{1}>\delta_{2}$, then:

(13) $$c>\frac{b}{\delta_{1}+\delta_{2}}$$

In our particular case, the condition of Equation (12) is clearly fulfilled since c<0 while b>0. Thus, the smaller gas will always be favored. In summary, it seems that the respective values of *b* and *c* should determine permeability and selectivity tendencies. These parameters are probably more determined by void size distributions, surface-to-volume ratios and void-to-void neck sizes rather than by the total fraction of free volume. Thus, they can be considered independent of the filler load if these factors can be attributed mostly to the polymer matrix, which should certainly be the case for low contents of PPN. 

By using the effective medium approximation described by Tena el al. [41], the effective permeability *P_eff_* of a medium constituted by a fraction *ϕ_d_* of a dispersed medium with permeability *P_d_* within a continuous medium of permeability *P_c_* in a fraction *ϕ _c_* = 1 − *ϕ_d_*, is given by:

(14) $$\phi_{d}\frac{P_{d}-P_{eff}}{P_{d}+2P_{eff}}+\phi_{c}\frac{P_{c}-P_{eff}}{P_{c}+2P_{eff}}=0$$

**Figure 7:**
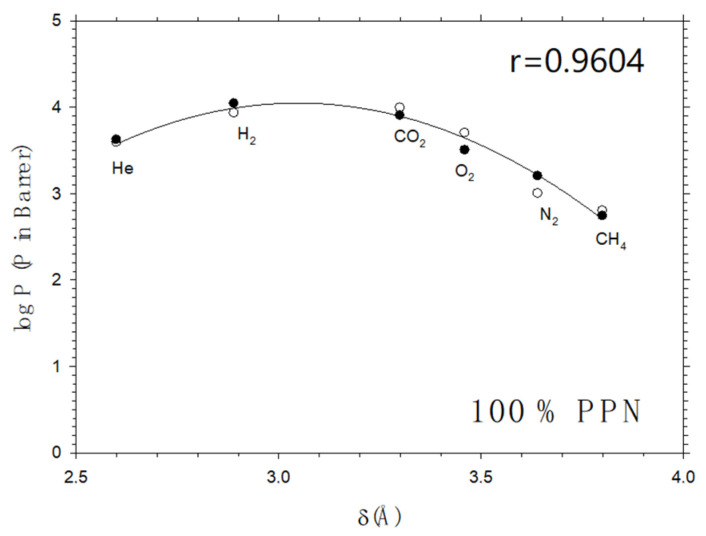
Permeability as a function of the gas kinetic diameters for pure PPN obtained by fitting the data for 6FCl-APAF series both before (hollow symbols) and after (filled symbols) thermal rearrangement. The line corresponds to the fitting to Equation (9).

This allows a fitting of the permeability of the corresponding MMMs versus the PPN fraction to extrapolate the pure PPN permeability (*P_d_*) for all the gases and polymer matrices before and after thermal rearrangement. In Figure 7, the corresponding results are shown in a plot similar to Figure 4 and Figure 5. Note that the corresponding fitting parameters cannot be used, as will be done in Section 5 for the relatively low filler fraction data, shown in Table 1 and Table 2, to get information on free volume changes, because it cannot be assumed that the *b* and *c* parameters could remain constant if the polymer matrix tended to disappear. 

**Figure 8:**
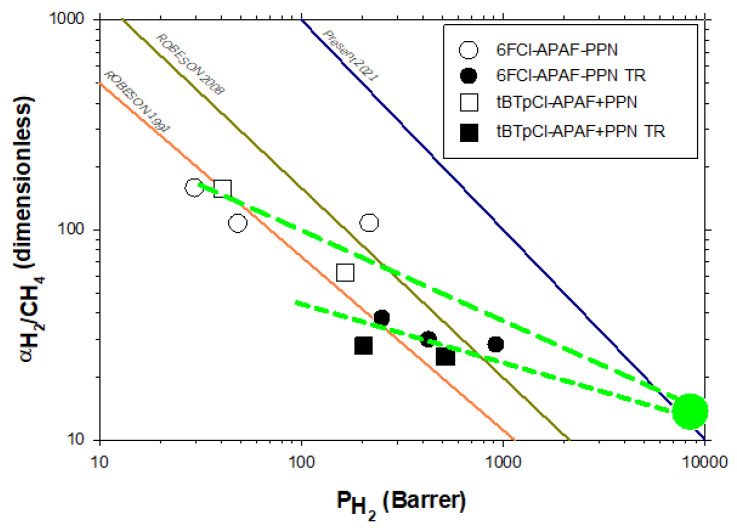
Selectivity versus permeability (Robeson’s plot) for H_2_/CH_4_ gas pair. The green symbol corresponds to the pure PPN permeability and selectivity obtained as explained in Figure 7. The green dashed lines correspond to approximate extrapolation of both the tBTpCl-APAF and 6FCl-APAF polymeric matrices. Both methods seem highly consistent.

The results for the pure filler would not depend on whether data corresponding to thermally rearranged or non-thermally rearranged MMMs were used, as far as we can assume that the PPN is not substantially affected by the thermal treatment. If these values of permeabilities are used to evaluate the pure PPN selectivity versus permeability, quite consistent results are obtained as shown, for example, for the H_2_/CH_4_ pair in Figure 8. Indeed, all results for selectivity versus permeability, before or after thermal rearrangement and for both the polymer matrices used here, extrapolate to the same pure PPN datum with a good approximation. This suggests that thermal rearrangement does not affect the PPN significantly as previously supposed.

**Figure 9:**
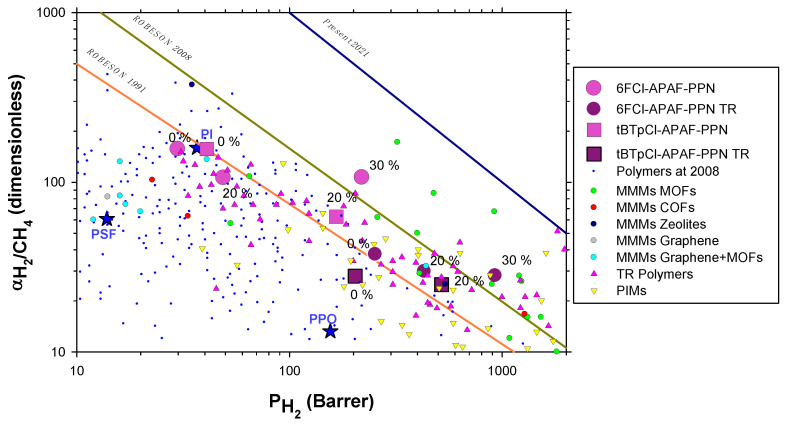
Selectivity versus permeability (Robeson’s plot) for H_2_/CH_4_ gas pair. Dark blue dots correspond to the cases used by Robeson to establish trade off lines [32,33]. Other colored symbols are described in the figure and correspond to TRs and PIMs [47] and to MMMs taken from Chuah et al. [42]. Stars correspond to some commercial membranes within the area shown: PSF polysulfone, PI polyimide and PPO poly(phenylene oxide)**.**

In Figure 8, the 1991 and 2008 trade-off lines are shown [32,33]. The additional line to the right represents the most well-received upper bound according to the corresponding literature to date [42,43,44,45]. For the sake of readability, we show separately in Figure 9 some results taken from the literature within the H_2_/CH_4_ Robeson plot, including the representative points used by Robeson to establish them [32,33]. Note that these results are quite good, as high permeabilities and selectivities are reached, overpassing the permeability versus selectivity 2008 trade-off line. These performances are only surpassed by some MMMs, including MOFs as fillers and some PIMs. Our precursor membranes have shown high selectivities and good permeabilities, and after thermal rearrangement they exhibited high permeabilities and relatively lower selectivities within the zone typical of PIMs without their time instabilities linked to aging and plasticization [46].

## 6. Free Volume Fraction as a Function of Filler Content

**Figure 10:**
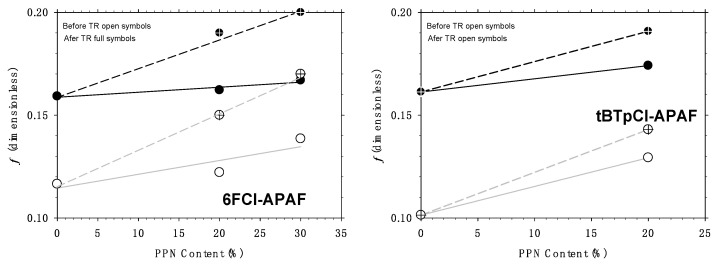
Changes in FFV as evaluated according to the procedure outlined in Section 2 (crossed symbols and dashed lines) and by the procedure outlined in Section 3 (non-crossed symbols and continuous lines). On the left the results for membranes with the 6FCl-APAF matrix and on the right for membranes with the tBTpCl-APAF matrix.

If we assume that *b* and *c* do not depend on the PPN content, the relative changes in *f* can be obtained from the values of both *bf* and *cf* of Table 1 and Table 2. The same results are obtained from *bf* as from *cf*, which indicates a good coherence of the procedure. This method leads to increases in the FFV as shown in Figure 10 compared with the results obtained by the method outlined in Section 3. 

Note that thermal rearrangement increases the FFV, which seems logical considering the increased stiffness of the chain after thermal rearrangement. Both before and after thermal rearrangement, the free volume increases when more PPN is present within the polymeric matrix. This increase is quite similar for both polymeric matrices. 

**Figure 11:**
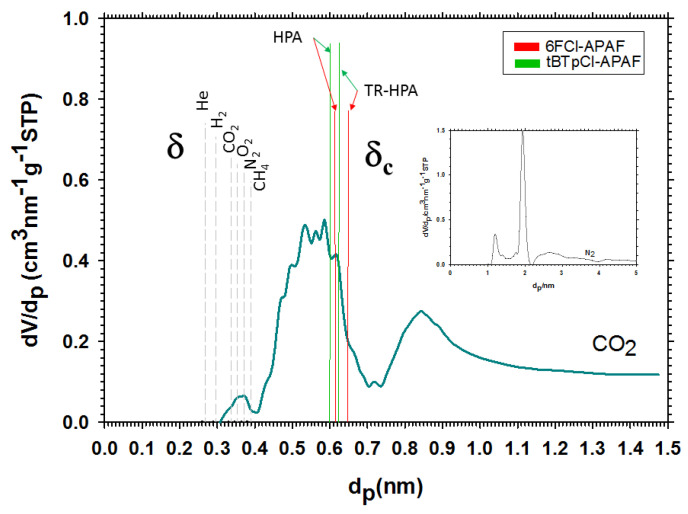
PPN pore size distribution as compared with mean chain segment lengths *δ_C_* and gas kinetic diameters *δ* for carbon dioxide and nitrogen (insert).

The procedure explained in Section 2 should give a good approximation to the actual free volume while that explained in Section 3 should give the permeability determining free volume. Thus, for both polymeric matrices it seems that not all the free volume present in the membrane is acting to increase permeability, possibly because some free volume would be trapped within the PPN particles, keeping it inaccessible. It is worth noting that, in all cases, the increment in the filler loads increases the percentage of inaccessible or trapped free volume, possibly by increasing the agglomeration of the filler. On the other hand, the phenomenon is slightly affected by thermal rearrangement. In any case, the fraction of trapped volume varies from 22 to 23% before thermal rearrangement and from 17% to 20% for thermally rearranged 6FCl-APAF. For tBTpCl-APAF, the fraction of trapped volume is 9% before thermal rearrangement and 11% after it. The fraction of trapped free volume seems to be lower for the tBTpCl-APAF matrix than for the 6FCl-APAF one, possibly because tBTpCl-APAF could approach PPN better. Indeed, the lower trapped free volume for the tBTpCl-APAF matrix could be correlated, for example, with its smaller mean chain length, *δ _c_* as detected by WAXS [14,23] as shown in Figure 11, where *δ* (the gas kinetic diameters of the gases used here) and *δ_c_* are compared with the pore size distribution of PPN as detected by adsorption-desorption isotherms. This could be explained in terms of the better penetration and compatibility of PPN and the tBTpCl-APAF matrix.

Pore characterization was carried out from N_2_ adsorption-desorption isotherms measured at −196 °C (77 K) in the volumetric device Autosorb iQ (Quantachrome Instruments) in the 10^−4^ to 0.995 relative pressure (*p*/*p_o_*) range. The samples were degassed at 120 °C for 10 h under vacuum, before the initiation of the sorption measurements, to eliminate possible adsorbed gases or water vapor. Pore size distributions were also obtained from the N_2_ isotherms by the non-local density functional theory equilibrium model (NLDFT). Acquisition and calculation were carried out by Quantachrome ASiQwin software (version 5.21). Pore characterization was also carried out from CO_2_ adsorption-desorption isotherms measured at 0 °C (273.15 K) at pressures up to 1 bar. 

It is apparent that CO_2_ detects smaller pores than N_2_, which is due to their quite different sizes. PPN pores seem to cover wide ranges with significant peaks at 0.35, 0.55, 0.85, 1.2, 2 and 2.5 nm. The kinetic diameters of the gases are well below the pore radii of PPN, which would mean that the presence of PPN would mainly increase diffusivity of all the gases studied. Finally, tBTpCl-APAF, having a lower average chain segment length, could enter (at least partially) into a higher fraction of the PPN pores. 

## 7. Conclusions

Diverse MMMs were presented in this work, which were manufactured based on two different HPA matrices (6FCl-APAF and tBTpCl-APAF) loaded with a PPN (prepared from triptycene and trifluoroacetophenone) in different percentages. The membranes were also thermally treated, inducing the transformation of HPA into polybenzoxazole (β-TR-PBO). The membranes were functionally characterized by analyzing the permeability and selectivity for He, H_2_, N_2_, O_2_, CH_4_ and CO_2_ gases.

From the analysis of the permeability as a function of the gas kinetic diameters, permeability decreases for gases with higher kinetic diameters regardless of the membrane, both in the case of precursor membranes and those thermally treated. Even though the membranes with each polymeric matrix showed the same behavior, membranes formed using the tBTpCl-APAF matrix exhibited higher permeabilities. In this work, a model for the permeability dependence on free volume fraction $P=A\;e^{Bf}$ has been proposed with *B* following a quadratic dependence (*B* = *a* + *b*δ + *c*δ^2^) on the kinetic diameter with *A*, a, *b* and *c* constants that should be related to the quality of the free volume (size distribution and/or the ratio of surface area to volume, etc.). This model seems coherent and fits our permeability versus kinetic diameter data well, allowing for the evaluation of free volume fractions determining permeability if *b* and *c* are assumed to be independent of the filler load (which seems reasonable for low loads). 

With regards to the analysis of the FFV with the PPN content, the free volume increases when the PPN content increases in both precursor and thermally treated membranes for both polymeric matrices. However, from a comparison of free volume fractions obtained from permeabilities and densities, it seems that not all the free volume existing in the membrane is acting to increase permeability, maybe due to the presence of inaccessible or trapped free volume. The fraction of trapped free volume, which increases with higher filler loads, seems to be lower for the tBTpCl-APAF matrix than for the 6FCl-APAF one, possibly because tBTpCl-APAF could access PPN pores better due to its smaller mean chain length that could fit better within the pore entrances of PPN. This phenomenon is slightly affected by thermal rearrangement.

In general, the higher the PPN content, the better the permeability versus selectivity properties of the membranes reported in this work. This improvement is quite notable for the membranes with the 6FCl-APAF matrix for the H_2_/CH_4_ gas pair, as they are located above the 2008 Robeson limit.

By using an effective medium approximation to extrapolate permeabilities to those of a pure filler, which cannot be actually measured, we have seen that for both thermally rearranged and non-rearranged MMMs, the pure filler value corresponds to the same tendency appearing in the Robeson’s plots for the MMMs with a low filler content, leading to selectivity versus permeability on the present upper bond.

## Figures and Tables

**Table 1:** Fitted parameters of Equation (10) for the membranes with 6FCl-APAF matrix. In this equation P was given in Barrer and δ in Å.

	PPN Loading	*lnA* + *af*	*bf*	*cf*
Before TR	0% PPN (r = 0.9886)	−25.8 ± 4.0	22.3 ± 2.5	−4.2 ± 0.4
	20% PPN (r = 0.9760)	−27.5 ± 5.4	23.4 ± 3.4	−4.3 ± 0.5
	30% PPN (r = 0.9793)	−31.8 ± 4.8	26.9 ± 3.1	−4.9 ± 0.5
After TR	0% PPN (r = 0.9557)	−28.8 ± 5.7	24.4 ± 3.6	−4.3 ± 0.6
	20% PPN (r = 0.9251)	−30.0 ± 6.2	25.3 ± 4.0	−4.4 ± 0.6
	30% PPN (r = 0.9251)	−30.6 ± 7.0	26.0 ± 4.4	−4.5 ± 0.7

**Table 2:** Fitted parameters of Equation (10) for the membranes with tBTpCl-APAF matrix. In this equation P was given in Barrer and δ in Å.

	PPN Loading	*lnA* + *af*	*bf*	*cf*
Before TR	0% PPN (r = 0.9886)	−14.6 ± 3.0	15.7 ± 1.5	−3.2 ± 0.3
	20% PPN (r = 0.9760)	−20.4 ± 4.4	19.5 ± 2.8	−3.7 ± 0.4
After TR	0% PPN (r = 0.9557)	−15.3 ± 2.7	15.6 ± 1.9	−3.0 ± 0.5
	20% PPN (r = 0.9251)	−18.9 ± 4.2	18.2 ± 3.2	−3.3 ± 0.4

## Data Availability

The raw and processed, data, and procedures required to reproduce these findings have been fully described in this paper and/or referenced. Additional data cannot be shared at this time due to technical limitations and are not needed to reproduce our results.
